# The Treatment of Joint Pain with Intra-articular Pulsed Radiofrequency

**DOI:** 10.5812/aapm.10259

**Published:** 2013-09-01

**Authors:** Pietro M Schianchi, Menno E Sluijter, Susan E Balogh

**Affiliations:** 1 S. Anna Clinic, Lugano, Switzerland; 2 Centre for Pain Medicine, Swiss Paraplegic Centre, Nottwil, Switzerland

**Keywords:** Osteoarthritis, Pulsed Radiofrequency reatment, Chemokines, Arthralgia, Allostasis, Joint Prosthesis

## Abstract

**Background:**

The intra-articular (IA) application of pulsed radiofrequency (PRF) for pain in small and large joints represents a recent development that has proven to be effective in many cases. We performed a retrospective study of 89 such procedures in 57 consecutive patients with chronic articular pain.

**Objectives:**

The aim of this retrospective study is to evaluate the effectiveness of intraarticular PRF in a group of 57 consecutive patients with chronic joint pain.

**Patients and Methods:**

Patients with intractable joint pain for more than 6 months were treated with IA PRF 40-45V for 10-15 min in small joints and 60V for 15 min in large joints using fluoroscopic confirmation of correct needle position. A total of 28 shoulders, 40 knees, 10 trapezio-metacarpal, and 11 first metatarso-phalangeal joints were treated. Results were evaluated at 1, 2, and 5 months. The procedure was repeated after 1 month in 10 patients with initial suboptimal results. Success was defined as a reduction of pain score by at least 50%.

**Results:**

All groups showed significant reductions in pain scores at all three follow-up visits. Success rates were higher in small joints (90% and 82%, respectively) than large ones (64% and 60%, respectively). Interestingly, IA PRF was successful in 6 out of 10 patients who had undergone previous surgery, including 3 with prosthetic joint replacement and in 6 of the 10 repeated procedures. There were no significant adverse effects or complications.

**Conclusions:**

IA PRF induced significant pain relief of long duration in a majority of our patients with joint pain. The exact mechanism is unclear, but may be related to the exposure of immune cells to low-strength RF fields, inducing an anti-inflammatory effect. The success rate appears to be highest in small joints. We recommend additional research including control groups to further investigate and clarify this method; our data suggest that it may represent a useful modality in the treatment of arthrogenic pain.

## 1. Background

Pulsed radiofrequency (PRF) has initially been used as an alternative to continuous RF in the treatment of nerves that transmit painful stimuli and for treating peripheral nerves that cause neuropathic pain (1-4). In 2006, a new application was developed; it was found that PRF could also effectively relieve discomfort from painful joints if the needle was placed within the joint (Teixeira A. 2006, personal communication, (5). The first report on this new method was published in 2008 (6).We have cautiously further explored the potential of this method. In this article, we report the results of 89 intra-articular PRF procedures in 57 consecutive patients who were treated according to the first publication.

### 1.1. Articular Pain

Articular pain is an important socioeconomic problem. For example, the prevalence of knee pain in the 40 to 79 year old community of the United Kingdom is about 25.3% (7). The exact etiology is not fully understood. Infectious, metabolic, autoimmune, traumatic, and, in particular, degenerative processes may all play a role, causing an initial inflammatory response that is characterized by increased local production of pro-inflammatory cytokines such as tumor necrosis factor (TNF)-α, interleukin (IL)-1β, and IL-6 (8, 9). Cytokines are small proteins produced by either immune (macrophages or helper T-cells) or non-immune cells (endothelial cells, Schwann cells, and their derivatives such as satellite cells in the dorsal root ganglion [DRG]). Under inflammatory conditions, cytokines are released and act on a number of different cells, serving as communicators and often as part of a cytokine cascade. Furthermore, pro-inflammatory cytokines maintain an up-regulated inflammatory response. There is increasing evidence that they also play an important role in the generation and maintenance of pain (10, 11) thus contributing to the loss of normal function in all phases of articular disorders. Joints are innervated by the articular branches of the nerves that supply the muscles acting upon them. Numerous simple nerve endings are located at the attachments of joint capsules and ligaments and are believed to be terminals of unmyelinated and thinly myelinated nociceptive axons. The articular nerves contain Aβ-, Aδ, and C-fibers. Free nerve endings have been found in all joint structures other than normal cartilage (12). The generation of pain from the inflammatory process is not simply due to conduction of nociceptive stimuli through the conventional channels. Although, research has shown that there is peripheral sensitization of primary afferent nociceptors in the joint itself, yet there is also a second relevant mechanism. Cytokines may also be transported to the DRG, crossing the blood-nervous-system barrier to reach the dorsal horn of the spinal cord, causing activation of microglia and astrocytes (13). This factor may potentially frustrate attempts to treat joint pain by ablation of afferent nerves. Osteoarthritis (OA) represents the most frequent form of joint disease, and is common in patients over 60 years of age. It has been associated with the allostatic load of the immune system (14), and indeed, mildly elevated C-reactive protein (CRP) levels have been demonstrated in patients with early OA (15). Especially in the early stages, OA produces pain and a reduced range of motion of the affected joint. The disease typically has a chronic, fluctuating course leading to changes in the structure of the synovia, cartilage, and subjacent bone. Experimental data have shown that OA is a result of an episode of mild inflammatory processes within the joint (16) that lead to high levels of pro-inflammatory cytokines (8-10, 17). Destruction of articular cartilage and remodelling of subchondral bone are prominent features of the later stages of the disease (18). Most studies on pain in OA have involved the knee joint. It has recently been suggested that magnetic resonance imaging (MRI) findings such as synovial hypertrophy, synovial effusions, and subchondral bone-marrow edema are found more frequently in painful than in non-painful knee joints with OA (19). The management of OA pain includes a myriad of conservative treatments with limited efficacy. Infiltrations with corticosteroids and viscosupplementation are widely used, but reportedly have short-lasting (20) or no effects; their use is often contraindicated, especially in small joints (19). The more recent method for treating OA by the means of anti-inflammatory drugs directed at cytokines to prevent the progression of structural changes of the joint has been disappointing for a number of reasons, and needs further investigation in regards to delivery form and reduction of toxicity (10). For many patients, the current treatment options for OA are thus unsatisfactory, and this leads to abuse of non-steroidal anti-inflammatory drugs, expensive conservative therapies, and repeated corticosteroid infiltrations. No clear consensus exists in regards to the timing and indications for major surgical procedures for OA (21).

### 1.2. PRF

Radiofrequency (RF) has been used for decades to heat the tip of an electrode to high (70 – 90 °C), destructive temperatures. The method is used in the treatment of chronic pain to destroy small nerves conducting nociceptive stimuli, for tumor ablation, and in cardiology to ablate abnormal atrioventricular connections. During PRF, RF current is delivered in short pulses. The recommended parameters are a pulse width of 20 msec and a rate of 2 Hz, but shorter pulse widths (5-10 msec) and frequencies of up to 5 Hz are also commonly used. During the pulse, the oscillating frequency is 420,000 Hz. Following PRF procedures there can be some mild tissue destructions around the electrode (22, 23), but this is not clinically detectable. The destruction is caused either by thermal effects at the tip during the pulse (“heat spikes”) or by high electric fields both at the tip and along the shaft. The mode of action of PRF is probably not thermal, because any significant rise in temperature during the pulse is limited to a distance of 0.1 mm beyond the tip (24), while any temperature rise on the cylindrical part of the electrode is negligible. The effect therefore appears to be triggered by the generated electric fields, which are very high, close to the tip of the electrode, but rapidly diminish with increasing distance from the tip. Electric fields are abundant around the cylindrical part of the tip, and extend much farther here because they are less rapidly dispersed.

## 2. Objectives

This retrospective study of 57 consecutive patients with chronic articular pain treated with intraarticular PRF aims to evaluate the effectiveness of this new technology and the duration of the benefit superior to the use of steroid infiltrations.

## 3. Patients and Methods

### 3.1. Patients

Fifty-seven consecutive patients with articular pain of the shoulder, knee, trapezio-metacarpal (TMC), and first metatarso-phalangeal (MP I) joints underwent 89 procedures with intra-articular PRF between January 2007 and July 2010. The material in this paper constitutes a retrospective review of our experience with intra-articular pulsed radiofrequency for these four types of joint pain. Written informed consent for the procedure as well as for use of the data was obtained from all cases, and the review was done in concordance with internationally accepted ethical principles. Since the study is retrospective, an exemption to IRB approval was granted by the local ethics committee. The basic demographics are shown in Table 1. There was a marked prevalence of females in the group with pain in large joints. In concordance with the literature, the prevalence of MP I joint pain was higher in our female patients, but the incidence of TMC joint pain was almost equal for both sexes. The pain present in patients mainly related to degenerative conditions of the joints due to primary OA. In 1 case MP I joint involvement was related to rheumatoid arthritis, but the disease was under control and not in an acute, florid stage. None of the other treated patients had an autoimmune disease, and none had sustained a recent trauma. Patients only qualified for treatment if they had had pain with a score of more than 3 on a numerical rating scale (NRS) of 0 – 10 for a period of at least 6 months. The number and location of the treated joints and the duration of pain before treatment are shown in Table 1. The average NRS score in our patient population was 8.15. Five patients with shoulder pain and 5 with knee pain had undergone a major joint operation. In particular, 2 patients had a shoulder and 1 had knee prosthesis. Steroid infiltrations had been performed elsewhere in 5 shoulders twice and in 4 knees once. The results had been disappointing, with improvement lasting for a few weeks at most. 

### 3.2. Clinical and Radiographic Data

Patients were questioned about previous trauma, professional and leisure activities, and daily habits. The history is obviously important in order to rule out metabolic, infectious, and autoimmune causes of pain. If there was any doubt as to the etiology, the patient was referred to a rheumatologist prior to PRF treatment. Numerous clinical tests can be employed to examine joints, especially the shoulder, knee, and hip. The tests used to examine our patients were the Mc Murray- and Steinmann I/II tests for knees; the shoulder-joint was checked with the Appley-test and performance of active and passive abduction, external rotation and adduction. The Grind-test was applied for TMC- and manual examination for MP 1- joints. A further important diagnostic procedure is the manual examination of the joint, which can indicate involvement of the surrounding ligaments and tendons, bursitis, or the presence of trigger points, which can mimic articular pain. Overall, 68.4% of the patients had undergone plain X-ray and/or MRI investigations. In all but a few cases with clear pain-producing pathology, the radiologic images were not helpful in determining which part of the joint was generating the pain.

### 3.3. Description of the Procedures

A NeuroTherm NT-1100 lesion generator was used. Following local anesthesia with 1% lidocaine, the PRF needle (SMK C-10, 22G, active tip 10 mm for large joints and SMK C-5, 22G, 5-mm active tip for small joints; NeuroTherm, Wilmington, MA) was inserted into the joint under fluoroscopic guidance in two planes. All procedures were performed under aseptic conditions in the operating room and following a standardized protocol to avoid unnecessary exposure to radiation. The mean X-ray exposure time was around 60 sec per joint.For shoulders a posterior or anterior approach and for knees a superior, medial, or lateral retropatellar approach was employed. The type of approach was chosen so that it enabled insertion of the PRF cannula as close as possible to the painful area within the joint. The small joints were entered using a “tunnel-vision” fluoroscopic approach, taking care to clearly visualize the intra-articular space. The lateral view is necessary for determining the depth of the needle in the joint. Intra-articular PRF was repeated in 5 shoulders, 4 knees, and 1 MP I joint in patients who had less than 50% pain relief after 1 month and who requested a second treatment. All other patients underwent a single treatment. The parameters that were used are shown in Table 2. 

### 3.4. Follow-up Protocol

Joint pain was evaluated at 1, 2, and 5 months after the PRF procedure. Patients with a successful outcome at 5 months were interviewed by telephone shortly before this article was prepared. The NRS scores and global perceived effect were evaluated on a 7-point Likert-scale. Success was defined as a reductionof NRS score by at least 50%.

**Table 1.** Demographic Data, Treated Joints and Mean Duration of Pain

	Knee	Shoulder	TMC ^a^	MP1 ^a^
**Female**	18	15	3	6
**Male**	8	2	4	1
**Mean age, y**	62.3	68.1	73.0	64.5
**Range**	33-91	50-81	69-81	47-74
**No. of treated**	40	28	10	11
**Pain duration, mo, mean**	22.5	23.7	14.5	60.2

^a^ Abbreviations: MP1, First Metatarso-Phalangeal;TMC, Trapezio-Meta-Carpal

**Table 2.** PRF Parameters

	Impedance, Ω, mean	Pulse width, msec	Frequency, Hz	Time, min	Temperature, °C,mean
**Large joints**	241.4	5-10	2-5	15	38.9
**Small joints**	573.5	5-10	2-5	10-15	32.2

## 4. Results 

**Figure 1.**
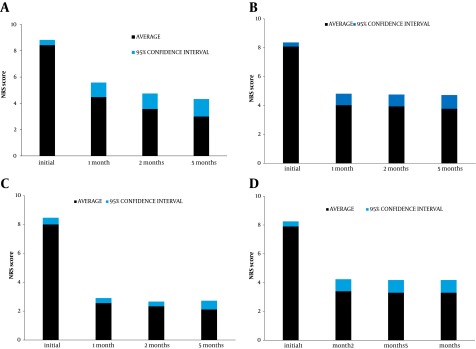
Mean NRS Scores With 95% Confidence Intervals A) Shoulder, B) Knee, C) TMC joint, D) MP1 joint

The results are given as incidence of success at the 5-month follow-up (Table 3) and average values of NRS and Likert scores up to the 5 months (Figure 1 and Table 3). All 10 repeated procedures were registered as failures despite the fact that 6 of these patients subsequently had a successful outcome. There was a significant reduction in NRS scores for all groups at the 1-month follow-up (Figure 1): P < 0.0001 for the patients with knee, MP I, and TMC joint treatment, P < 0.05 for shoulder treatment. At 3 months, the result in the shoulder group had improved further (P < 0.0001). Differences for 1- and 5-month follow-up (3- to 5-month for shoulders) were not significant. The small joints had a significantly better outcome than the knees and shoulders together (P = 0.007). The intra-articular PRF procedure was successful in 2 out of 5 patients with previously operated shoulders (both with joint prostheses) and 4 out of 5 with operated knees (including 1 prosthesis), and was successful and long-lasting in 3 out of 5 shoulders and 3 out of 4 knees with previous unsatisfactory steroid infiltrations. 

### 4.1. Complications

The procedure was well tolerated, with minimal to no postoperative discomfort during the first few days. No complications have been observed.

### 4.2. Follow-up After 5 Months

Table 4 shows the follow-up periods after the PRF treatment. The data from the 6 patients were not available for interview by telephone, because they could not be traced or because of incidental events (shoulder fracture, operation for a meniscus lesion). In the shoulder and knee patients there was a tendency for further improvement after 5 months. This was not the case after treatment of the small joints. 

**Table 3.** Success Rates Based on the NRS and Average Likert Scores (The Range was 4–7 for All Values)

	Knee	Shoulder	TMC ^a^	MP1 ^a^
**Total**	40	28	10	11
**Initial NRS , No.** ^**a**^ **, No.**	8.08	8.43	7.90	7.91
**Success, No.**	25	18	9	9
**Success, %**	62.5	64.3	90.0	81.8
**Likert scores**				
1 month	5.68	5.64	6.22	5.8
3 months	5.68	5.52	6.44	5.9
5 months	5.89	6.10	6.56	5.8

^a^Abbreviations: MP1, First Metatarso-Phalangeal;NRS, Numeral Rating Scale;TMC, Trapezio-Meta-Carpal

**Table 4.** Mean Follow up Periods After PRF Treatment

	Period, mean
**Shoulder**	13 months (5–26)
**Knee**	16 months (5–47)
**TMC ** ^**a**^	8 months (5–13)
**MP1 ** ^**a**^	9 months (5–15)

^a^Abbreviations: MP1, First Metatarso-Phalangeal; TMC, Trapezio-Meta-Carpal

## 5. Discussion 

This is a retrospective study. All procedures were performed in the practice of the first author, and the organizational environment did not permit comparison of the data to a control group. Conclusions should thus be drawn with reservations. Nevertheless, the results are encouraging because they did not in anyway contradict our first impression (6) or a previous report regarding the method (25). Both the success rate and the relatively long duration of action of intra-articular PRF without adverse side effects suggest that this may represent a promising option for the treatment of refractory joint pain. The apparent effectiveness of this method has caused a fundamental change in our concept of the mode of action of PRF. Prior to Teixeira’s initial observation, any theory as to the mode of action of PRF involved the nervous system (26, 27). In the intra-articular technique, however, the tip of the cannula lies within a space surrounded by synovium, cartilage, and bone. In large joints such as the knee, the articular capsule, which is richly innervated, is quite distant from the tip of the cannula, so that nerves are not in close proximity. For this reason, a direct influence by the electric field on pain-generating nerves would appear to be unlikely. We therefore postulate that electric fields from the low range of the spectrum may influence the production of pro-inflammatory cytokines, which are the same in bone, cartilage, synovium, and the immune system. While the hypothesis of a biological effect of low-range electric fields is not new – experiments comparing the effects of PRF to continuous RF at the same tip temperature clearly indicate that such effects are a reality (22, 28) – yet the effect not being confined to neurons, incorporating immune cells as well, is a new concept. This would be in accordance with many anecdotal reports of decreases in serum CRP levels following intra-articular PRF. With regard to cytokine levels, while intra-articular PRF could have an effect that is comparable to that of steroid infiltration, the paths leading to that effect might be diametrically opposed. It would seem unlikely that PRF would provoke an immediate anti-inflammatory response in a cell when the latter is exposed to the molecule-jarring effect of the alternating electric fields. A brief inflammatory phase would be much more logical, and early laboratory work on the subject indicates that this is exactly what may happen (29). We therefore recommend that PRF and steroid infiltrations not be combined until this matter has been clarified and more well-informed judgment is possible. We find it remarkable that all 3 patients who had a joint prosthesis had a favorable outcome; this is, however, in concordance with several reports that have reached us from other colleagues. Persisting pain following joint replacement may of course have local causes, but it is difficult to imagine how PRF could have any influence on this type of pain. We suspect that in some of these patients persisting pain is a manifestation of allostatic load rather than a local orthopedic problem. We used fluoroscopic guidance to ensure smooth, painless, and precise introduction of the PRF cannula into the joint without complications. We prefer this method in small as well as large joints. Without the use of fluoroscopy, the incidence of unintended periarticular injection in finger joints is estimated at 23% (30). The use of ultrasonographic guidance is another option, but this requires extensive experience and frequent practice; furthermore, the maintenance of sterility during the procedure, particularly when smaller joints are involved, can be difficult. We have found that intra-articular PRF induced significant relief of pain in a majority of our patients with OA. The results were better in small joints than in large ones. The duration of pain relief was much longer than would have been expected from steroid injections according to the literature. We therefore recommend that further research comparing the effects of this method to a control series be undertaken.We have postulated that the effect of intra-articular PRF is caused by the exposure of immune cells to low-strength RF fields (31, 32). We recommend that PRF not be combined with steroid infiltration until the exact nature of this mechanism has been clarified. Considering the minimally invasive nature of this method, the absence of major complications, and the encouraging success rate in these and other patients with articular pain resistant to other therapies, intra-articular PRF may represent an additional therapeutic option for refractory joint pain.
